# Genetic variations of nucleoprotein gene of influenza A viruses isolated from swine in Thailand

**DOI:** 10.1186/1743-422X-7-185

**Published:** 2010-08-09

**Authors:** Nattakarn Thippamom, Donreuthai Sreta, Pravina Kitikoon, Roongroje Thanawongnuwech, Yong Poovorawan, Apiradee Theamboonlers, Kamol Suwannakarn, Sujira Parchariyanon, Sudarat Damrongwatanapokin, Alongkorn Amonsin

**Affiliations:** 1 Emerging and Re-emerging Infectious Diseases in Animals, Research Unit, Faculty of Veterinary Science, Chulalongkorn University, Bangkok, 10330, Thailand; 2 Center of Excellence in Clinical Virology, Faculty of Medicine, Chulalongkorn University, Bangkok, 10330, Thailand; 3 National Institute of Animal Health, Department of Livestock Development, Bangkok, Thailand

## Abstract

**Background:**

Influenza A virus causes severe disease in both humans and animals and thus, has a considerably impact on economy and public health. In this study, the genetic variations of the nucleoprotein (NP) gene of influenza viruses recovered from swine in Thailand were determined.

**Results:**

Twelve influenza A virus specimens were isolated from Thai swine. All samples were subjected to nucleotide sequencing of the complete NP gene. Phylogenetic analysis was conducted by comparing the NP gene of swine influenza viruses with that of seasonal and pandemic human viruses and highly pathogenic avian viruses from Thailand (n = 77). Phylogenetic analysis showed that the NP gene from different host species clustered in distinct host specific lineages. The NP gene of swine influenza viruses clustered in either Eurasian swine or Classical swine lineages. Genetic analysis of the NP gene suggested that swine influenza viruses circulating in Thailand display 4 amino acids unique to Eurasian and Classical swine lineages. In addition, the result showed 1 and 5 amino acids unique to avian and human lineages, respectively. Furthermore, nucleotide substitution rates showed that the NP gene is highly conserved especially in avian influenza viruses.

**Conclusion:**

The NP gene sequence of influenza A in Thailand is highly conserved within host-specific lineages and shows amino acids potentially unique to distinct NP lineages. This information can be used to investigate potential interspecies transmission of influenza A viruses. In addition, the genetic variations of the NP gene will be useful for monitoring the viruses and preparing effective prevention and control strategies for potentially pandemic influenza outbreaks.

## Background

Influenza A virus poses a serious threat to public health worldwide, particularly the virus circulating in humans and animal species such as birds, pigs and horses. Influenza A subtypes H1-3 and N1-2 have been circulating in the human population, while Influenza A subtypes H1 and 3 and N1-2 have been reported in swine. On the other hand, all H1-16 and N1-9 can be found in avian species [1,2]. The virus genome contains 8 segments of single-stranded RNA that encode 10-11 proteins. Among those genes, the NP gene plays a major role with regard to host range or host species barriers for influenza A virus [3-5]. Genetic analysis of the NP gene has facilitated identification of particular amino acids correlated with host specificity [6]. At least two large classes of NP gene, human and non-human, had been classified by phylogenetic analysis [3,7,8]. NP protein functions include encapsidation of the virus genome for RNA transcription, replication and packaging [9], interaction with polypeptides in nuclear localization signals [10], direct interaction with viral polymerase for unprimed viral replication [11] and cytotoxic T lymphocyte activation [12,13].

Recently, an influenza virus originating from swine (S-OIV 2009) has emerged in humans and subsequently spread worldwide. The 8 gene segments of the pandemic (H1N1) 2009 virus originated from human lineage (PB1), avian lineage (PB2, PA), Eurasian swine lineage (NA, M) and classical swine lineage (HA, NP, NS) [14,15]. This serves as an example that certain influenza A strains can harbor an NP gene that might not be host specific, such as the S-OIV in humans. The NP gene of S-OIV has been suggested to originate from the classical swine influenza virus.

As of April 2010, approximately 166 nucleotide sequences of the NP gene of influenza A viruses from Thailand have been reported to the public database (NCBI Influenza Virus Database). Among these 166 sequences, 97 were from avian (H5N1 = 96 and H3N2 = 1), 55 from human (H1N1 = 24, H3N2 = 22, and H5N1 = 9) and 14 from swine (H1N1 = 1, H1N2 = 1, and H3N2 = 6) viruses. In addition, most of the 166 sequences originated from virus isolated between 2000 and 2009, except for one virus that had been isolated in 1976. Due to the limited information on the NP gene of influenza viruses recovered from various species especially swine in Thailand, the objective of this study was to determine the genetic variation of the NP gene of influenza viruses isolated from swine in Thailand. In addition, the NP gene sequences of seasonal and pandemic 2009 human viruses as well as highly pathogenic avian influenza were retrieved from the database and included in the analysis.

## Results

### Complete NP gene of Thai swine influenza viruses

During 2005-2009, 12 swine influenza viruses were isolated from areas of intensive swine farming in central and eastern regions of Thailand. The 12 swine influenza isolates were identified as subtypes H1N1 (n = 6), H1N2 (n = 1) and H3N2 (n = 5) based on RT-PCR using subtype specific primers. To study the genetic variation of the viruses, nucleotide sequencing was performed on the complete NP gene of 12 swine influenza isolates. The resulting sequences were submitted to the GenBank database under accession numbers HM142746-HM142757. Virus characteristics and GenBank accession numbers of NP gene sequences are shown in table 1. In addition, the NP gene sequences of Thai avian (n = 25), human (n = 25), and swine (n = 14) influenza viruses retrieved from the public database (GenBank) were included in the analysis (Table 1).

**Table 1:** Influenza A isolates from human, swine and avian hosts used in this study.

Virus	Subtype	Year	GenBank #	Lineage
**Equine virus**				
A/Equine/Prague/1/56	H7N7	1956	M63648	
				
**Avian virus**				
A/Chicken//Thailand/CU-K2/04	H5N1	2004	AY590579	Avian
A/Duck/Thailand/71.1/04	H5N1	2004	AY651496	Avian
A/Goose/Thailand/79/04	H5N1	2004	AY651497	Avian
A/Chicken/Thailand/CU-23/04	H5N1	2004	AY770996	Avian
A/Chicken/Thailand/73/04	H5N1	2004	DQ076203	Avian
A/Chicken/Thailand/CK-160/05	H5N1	2005	DQ334761	Avian
A/Quail/Thailand/QA-161/05	H5N1	2005	DQ334769	Avian
A/Chicken/Thailand/CK-162/05	H5N1	2005	DQ334777	Avian
A/Chicken/Thailand/NIAH108192/05	H5N1	2005	AB450586	Avian
A/Chicken/Thailand/PC-170/06	H5N1	2006	DQ999891	Avian
A/Chicken/Thailand/PC-168/06	H5N1	2006	DQ999883	Avian
A/Chicken/Thailand/NP-172/06	H5N1	2006	DQ999877	Avian
A/Watercock/Thailand/CU-334/06	H5N1	2006	EU616887	Avian
A/Quail/Thailand/CU-330/06	H5N1	2006	EU616855	Avian
A/Duck/Thailand/KU-56/07	H5N1	2007	EU221252	Avian
A/Duck/Thailand/CU-328/07	H5N1	2007	EU616839	Avian
A/Duck/Thailand/CU-329/07	H5N1	2007	EU616847	Avian
A/Chicken/Thailand/NS-341/08	H5N1	2008	EU850417	Avian
A/Chicken/Thailand/NS-342/08	H5N1	2008	EU850425	Avian
A/Chicken/Thailand/NS-339/08	H5N1	2008	EU620657	Avian
A/Chicken/Thailand/PC-340/08	H5N1	2008	EU620665	Avian
A/Chicken/Thailand/ST-351/08	H5N1	2008	FJ868015	Avian
A/Chicken/Thailand/CU-354/08	H5N1	2008	CY047458	Avian
A/Chicken/Thailand/CU-355/08	H5N1	2008	CY047462	Avian
A/Duck/Thailand/AY-354/08	H3N2	2008	FJ802402	Avian
				
**Human virus**				
A/Thailand/5-KK-494/04	H5N1	2004	AY627889	Avian
A/Thailand/2-SP-33/04	H5N1	2004	AY627895	Avian
A/Thailand/1-KAN-1/04	H5N1	2004	AY626145	Avian
A/Thailand/676/05	H5N1	2005	DQ360840	Avian
A/Thailand/NK165/05	H5N1	2005	DQ372594	Avian
A/Thailand/CU23/06	Seasonal H3N2	2006	FJ912940	Human
A/Thailand/CU32/06	Seasonal H1N1	2006	FJ912910	Human
A/Thailand/CU46/06	Seasonal H3N2	2006	FJ912922	Human
A/Thailand/CU51/06	Seasonal H1N1	2006	FJ912928	Human
A/Thailand/NBL1/06	H5N1	2006	GQ466183	Avian
A/Thailand/CU280/07	Seasonal H3N2	2007	FJ912964	Human
A/Thailand/CU282/07	Seasonal H3N2	2007	FJ912970	Human
A/Thailand/CU356/08	Seasonal H3N2	2008	FJ912977	Human
A/Thailand/CU370/08	Seasonal H3N2	2008	FJ912985	Human
A/Thailand/CU1103/08	Seasonal H3N2	2008	FJ913012	Human
A/Thailand/CU-B4/09	Seasonal H3N2	2009	GQ902794	Human
A/Thailand/CU-B42/09	Seasonal H1N1	2009	GQ902802	Human
A/Thailand/102/09	Pandemic H1N1	2009	GQ166232	Classical swine
A/Thailand/104/09	Pandemic H1N1	2009	GQ169385	Classical swine
A/Thailand/CU-B5/09	Pandemic H1N1	2009	GQ866952	Classical swine
A/Thailand/CU-H9/09	Pandemic H1N1	2009	GQ866960	Classical swine
A/Thailand/CU-H106/09	Pandemic H1N1	2009	GQ866932	Classical swine
A/Thailand/CU-H276/09	Pandemic H1N1	2009	GQ866933	Classical swine
A/Thailand/CU-H340/09	Pandemic H1N1	2009	GQ866934	Classical swine
A/Thailand/CU-B938/09	Pandemic H1N1	2009	GQ866935	Classical swine
				
**Swine influenza virus**				
A/Swine/Thailand/KU5.1/04	H3N2	2004	FJ561061	Classical swine
A/Swine/Thailand/NIAH1481/00	H1N1	2000	AB434289	Eurasian swine
A/Swine/Thailand/NIAH550/03	H1N1	2003	AB434297	Eurasian swine
A/Swine/Thailand/NIAH9469/04	H1N1	2004	AB434305	Eurasian swine
A/Swine/Thailand/NIAH977/04	H1N1	2004	AB434313	Eurasian swine
A/Swine/Thailand/NIAH589/05	H1N1	2005	AB434321	Eurasian swine
A/Swine/Thailand/NIAH587/05	H1N1	2005	AB434329	Eurasian swine
A/Swine/Thailand/NIAH13021/05	H1N2	2005	AB434337	Eurasian swine
A/Swine/Thailand/NIAH-NW/03	H3N2	2003	AB434345	Classical swine
A/Swine/Thailand/NIAH464/04	H3N2	2003	AB434353	Eurasian swine
A/Swine/Thailand/NIAH586-1/05	H3N2	2005	AB434361	Classical swine
A/Swine/Thailand/NIAH59/04	H3N2	2004	AB434369	Eurasian swine
A/Swine/Thailand/NIAH874/05	H3N2	2005	AB434377	Classical swine
A/Swine/Thailand/NIAH101942/08	H1N1	2008	AB514939	Eurasian swine
				
**Swine virus characterized in this study**				
A/Swine/Thailand/CB-HF6/05	H1N1	2005	HM142750	Eurasian swine
A/Swine/Thailand/06CB2/06	H1N1	2006	HM142751	Eurasian swine
A/Swine/Thailand/CU-CB1/06	H1N1	2006	HM142752	Eurasian swine
A/Swine/Thailand/CS-K1/08	H1N1	2008	HM142753	Eurasian swine
A/Swine/Thailand/CU-CBP18/09	H1N1	2009	HM142754	Eurasian swine
A/Swine/Thailand/CU-CHL2/09	H1N2	2009	HM142755	Eurasian swine
A/Swine/Thailand/CB-NIAH586/05	H3N2	2005	HM142746	Classical swine
A/Swine/Thailand/NP-NIAH586-2/05	H3N2	2005	HM142747	Classical swine
A/Swine/Thailand/CS-NIAH586-3/05	H3N2	2005	HM142748	Classical swine
A/Swine/Thailand/NIAH586-4/05	H3N2	2005	HM142749	Classical swine
A/Swine/Thailand/CB-S1/05	H3N2	2005	HM142756	Eurasian swine
A/Swine/Thailand/CU-CB8.4/07	H3N2	2007	HM142757	Eurasian swine

### Phylogenetic analysis

**Figure 1:**
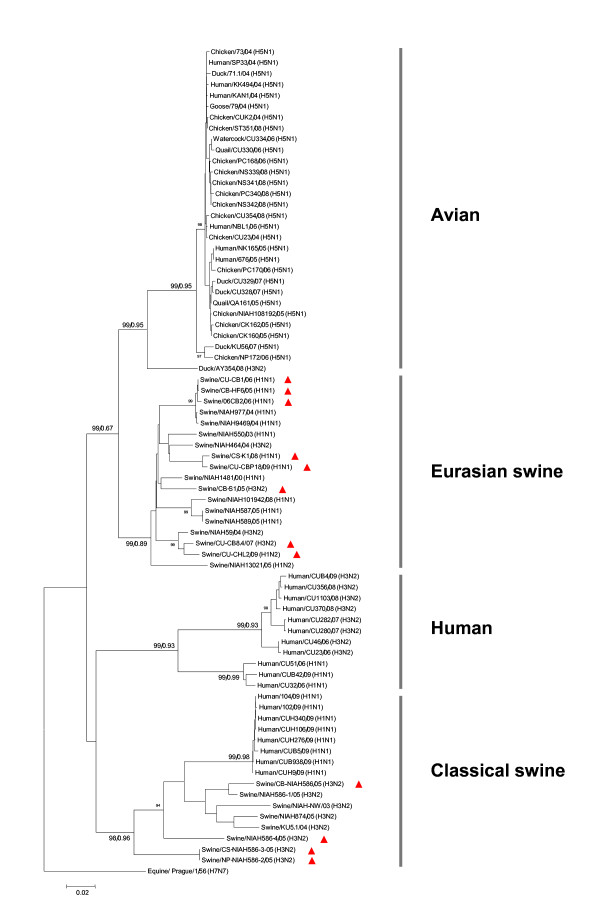
**Phylogenetic tree of NP gene of influenza viruses recovered from swine, human and avian hosts in Thailand**. The trees were generated using MEGA 4.0 applying the neighbor-joining algorithm. Tree topology was supported with bootstrap analysis with 1000 replicates and posterior probability from BMCMC analysis (Bootstrap, posterior probability). The swine influenza viruses characterized in the study are presented as triangles.

Phylogenetic analysis of 76 different NP nucleotide sequences of human (n = 25), avian (n = 25), swine (n = 14) Thai isolates and one reference NP nucleotide sequence of equine (n = 1) virus showed that the viruses clustered in distinct lineages represented by the avian, human, classical swine and Eurasian swine lineages (Fig 1). The avian NP lineage contains all avian influenza virus subtypes H5N1 (n = 24) and H3N2 (n = 1). In addition, all human H5N1 viruses (n = 6) also clustered in this avian NP lineage. A human NP lineage comprises two groups of seasonal human influenza subtypes H3N2 (n = 8) and H1N1 (n = 3). In contrast, the pandemic 2009 influenza subtype H1N1 (n = 8) clustered with the classical swine NP linage. The swine influenza viruses can be divided into 2 distinct lineages, Eurasian swine lineage and classical swine lineage. Based on topology of the phylogenetic tree, the Eurasian swine lineage is closely related to the avian lineage and had been previously designated "avian-like swine lineage" [3,16]. Eighteen swine virus subtypes H1N1, H1N2 and H3N2 from 2000-2009 clustered in this Eurasian swine lineage. On the other hand, 8 swine virus subtypes H3N2 and H1N1 were grouped with the classical swine lineage. It is noteworthy that 12 swine viruses characterized in this study clustered in both the Eurasian (H1N1 = 5, H1N2 = 1, and H3N2 = 2) and classical swine lineage (H3N2 = 4) (Table 1 and Fig 1). It should be noted that Thailand has imported swine for breeding from both Europe and North America. In general, phylogenetic analysis of NP gene sequences of influenza A viruses indicated that the NP gene is highly conserved and largely grouped within the host range of the respective virus.

### Genetic analyses

Pair-wise NP gene sequence comparisons of swine influenza viruses with 5 representative influenza viruses of equine (PR/56), avian (CUK2), human (CU32), Eurasian swine (9469/04) and classical swine lineages (K5/04) are shown in table 2. The Thai swine influenza viruses were found similar to 2 distinct lineages, the Eurasian and classical swine lineages. Eight swine influenza viruses displayed a high percentage of nucleotide identity (93.5-99.7%) to the European swine lineage (9469/04). On the other hand, 4 swine influenza viruses were similar to the classical swine lineage (K5/04) with 90.5-93.6% nucleotide identity. The deduced amino acids of the NP genes of 77 influenza viruses were compared to evaluate the host-specific nature of the NP gene. Few amino acid differences between lineages were detected indicating the highly conserved nature of the NP gene especially, in the avian lineage (table 3). Various reports have documented that particular amino acids are unique to distinct NP lineages [3]. In this study, one amino acid at position 105 was found correlated with the avian specific lineage (105V). In the human lineage, 5 amino acids at positions 16 (16D), 283 (283P), 293 (293K), 372 (372D), and 422 (422K) were highly conserved as human-specific amino acids. Moreover, some amino acids at positions 31, 33, 61, 100, 109, 136, 214, 377, and 455 showed potentially human-specific characteristics even though such amino acids can be found in either avian or swine lineages (Table 3). Four amino acids unique to Eurasian and classical swine lineages were identified at positions 350 (350K/T), 371 (V/M), 444 (V/I), and 456 (L/V). It should be noted that amino acids potentially unique to the pandemic H1N1 2009 were found at positions 100 (100I), 217 (217V), 313 (313V), 316(316M) and 425 (425V).

**Table 2:** Pair-wise sequence comparison of complete NP gene nucleotide sequences of 12 swine viruses and those of reference viruses

Virus/year (subtype)	Host	Lineage	Reference viruses				
			
			Equine	Avian	Human	Eurasian swine	Classical Swine
			
			PR/56	CUK2	CU32	9469/04	K5/04
PR/56 (H7N7)	Equine	-	100	83.7	81.9	83.8	82.4
CUK2/04 (H5N1)	Avian	Avian	83.7	100	82.7	88.9	82.3
CU32/06 (sH1N1)	Human	Human	81.9	82.7	100	82.6	84.1
102/09 (pH1N1)	Human	Classical swine	82.4	82.3	84.0	83.2	91.1
9469/04 (H1N1)	Swine	Eurasian swine	83.8	88.9	82.6	100	82.9
HF6/05 (H1N1)*	Swine	Eurasian swine	83.8	88.8	82.7	99.7	83.0
06CB2/06 (H1N1)*	Swine	Eurasian swine	83.8	88.7	82.7	99.5	83.1
CU-CB1/06 (H1N1)*	Swine	Eurasian swine	83.9	88.9	82.8	99.7	83.1
CS-K1/08 (H1N1)*	Swine	Eurasian swine	84.2	88.2	82.5	93.5	82.5
CU-CBP18/09 (H1N1)*	Swine	Eurasian swine	84.1	88.4	82.3	93.4	82.1
CU-CHL2/09 (H1N2)*	Swine	Eurasian swine	83.2	88.8	82.3	94.4	83.0
CU-CB8.4/07 (H3N2)*	Swine	Eurasian swine	83.6	89.2	82.6	94.3	82.9
CB-S1/05 (H3N2)*	Swine	Eurasian swine	84.5	89.0	82.8	95.0	82.1
K5/04 (H3N2)	Swine	Classical swine	82.4	82.3	84.1	82.9	100
CB-NIAH-586/05 (H3N2)*	Swine	Classical swine	82.6	82.9	83.5	85.1	93.6
NP-NIAH-586-2/05 (H3N2)*	Swine	Classical swine	82.7	85.1	82.5	87.1	91.2
CS-NIAH-586-3/05 (H3N2)*	Swine	Classical swine	82.7	85.1	82.5	87.1	91.2
NIAH586-4/05 (H3N2)*	Swine	Classical swine	82.5	83.4	82.7	85.7	90.5

* The Thai swine viruses characterized in this study

**Table 3:** Analysis of unique amino acids for avian, human, classical swine and Eurasian swine lineages.

Host	Lineage	n	Deduced amino acid position of NP protein															
			
			Human lineage														Avian lineage	
			
			16	31	33	61	100	109	136	214	283	293	372	377	422	455	105	450
Equine	-	1	G	K	V	I	R	I	L	K	L	R	E	N	R	D	I	N 1
Avian	Avian	25	G	R	V	I	R	I	L	R	L	R	E	N24/S11	R	D	V	S 24/G1
Human (H5N1)	Avian	6	G	R	V	I	R	I	L	R	L	R	E	N	R	D	V	S 6
Human (Seasonal)	Human	11	D	K8/R3	I	L	V	V	I	K	P	K	D	S9/G2	K	E10/D	M8/V3	S 5/G6
Human (Pandemic)	Classical swine	8	G	R	I	I	I	I	I	R	L	R	E	N	R	D	M	S
Swine (Eurasian)	Eurasian swine	18	G	R	V	I15/M3	R	I16/V2	L	R15/K3	L	R	E	V15/I3	R	D	M17/I 1	S12/N5/G1
Swine (Classical)	Classical swine	8	G	R	V6/I2	I	V6/R2	I	I6/L2	K7/R	L	R	E	N	R	D	M	N7/R

**Host**	**Lineage**	**n**	**Swine lineage**															
			
			**217**	**289**	**313**	**316**	**350**	**357**	**371**	**373**	**384**	**400**	**425**	**433**	**444**	**456**		

Equine	-	1	I	Y	F	I	T	Q	M	T	R	K	I	N	I	V		
Avian	Avian	25	I24/M	Y	F	I	T	Q	M	A24/T1	R	R	I	T	I	V24/A1		
Human (H5N1)	Avian	6	I	Y	F	I	T	Q5/K	M	A	R	R	I	T	I	V		
Human (Seasonal)	Human	11	I3/S8	Y	Y	I	T	K	M	N8/A3	R	R	I	T	I	V		
Human (Pandemic)	Classical swine	8	V	H	V	M	K	K	V	T	G8/R3	K	V	N	V	L		
Swine (Eurasian)	Eurasian swine	18	I	Y	F16/L2	I	T	Q17/K1	M	T	K	R17/K1	I	T17/N1	I	V		
Swine (Classical)	Classical swine	8	I7/V	H6/Y2	F	I	K	K	V	A	R	K	I7/V	N	V	L		

### Nucleotide substitution rate of the NP gene

Nucleotide substitution rates of the NP gene in swine, human and avian lineage viruses were calculated using BEAST v1.4.7 applying the Bayesian Markov Chain Monte Carlo (BMCMC). In this study, the nucleotide substitution rates of the NP gene in both Eurasian and classical swine lineages viruses were high, amounting to 2.92 × 10^-3 ^and 2.98 × 10^-3^, respectively. In addition, all human lineages (seasonal H1N1, H3N2 and pandemic H1N1) also displayed high nucleotide substitution rates of the NP gene (Table 4). On the other hand, the substitution rate of the NP gene in avian viruses was half (1.57 × 10^-3^) that of swine and human lineages, indicating the highly conserved nature or genetically static stage of the NP gene of avian viruses compared to human and swine viruses.

**Table 4:** Nucleotide substitution rates of NP gene of swine, human and avian influenza viruses in Thailand.

	n	Mean Substitution Rate (×10^-3^)	Substitution Rate HPD (×10^-3^)
Avian H5N1	91	1.57	0.92-2.22
Eurasian Swine	18	2.92	1.87-3.97
Classic Swine	8	2.98	1.56-4.30
Human Seasonal H1N1	14	2.11	1.32-2.88
Human Seasonal H3N2	22	2.56	0.69-4.40
Human Pandemic H1N1	8	2.57	1.79-3.21

## **Discussion**

In this study, we determined the NP gene sequences of 12 Thai swine influenza virus subtypes (H1N1 and H3N2) recovered between 2005 and 2009. Previous reports have provided some NP gene sequences of swine influenza viruses from Thailand [17,18]. However, none of those NP gene sequences has been comprehensively characterized. Since only 14 NP nucleotide sequences of Thai swine viruses have been stored at the public database, the results obtained from this study could help add significant information on swine influenza viruses in Thailand.

Phylogenetic analysis of the NP gene of 76 selected influenza viruses from Thailand and one representative for the NP gene (A/Equine/Prague/1/56 (H7N7) confirmed distinct clusters of the NP gene as equine, avian, human, European swine and classical swine lineages (Fig 1). The NP gene of influenza viruses has been distinguished into human and non-human groups [6-8]. Host specific NP groups including equine 1, recent equine, human-classical swine, H13 gull and avian differentiated by both RNA hybridization and phylogenetic analysis have been reported in previous studies [3,5]. Avian-like swine (Eurasian swine) and classical swine lineages have also been documented [19]. The result of this study confirmed that the NP gene is highly conserved within host-specific lineages. Most avian, human and swine viruses in Thailand cluster within their specific host ranges. For example, all avian influenza viruses as well as human H5N1 viruses cluster in the avian lineage, while seasonal human H1N1 and H3N2 are grouped with a separate human lineage. It should be noted that avian H5N1 viruses have been isolated from several mammalian species such as humans, tigers, cats, dogs and possibly other domestic animals. However these H5N1 viruses displayed avian characteristics and were grouped with the avian linage [20-22]. In addition, several studies have reported that the NP gene of pandemic H1N1 2009 displays classical swine characteristics [14,15]. Evidence of the pandemic H1N1 2009 human viruses displaying a swine-like NP gene and of H5N1 human viruses containing an avian NP gene has suggested that the NP gene can be utilized for tracing interspecies transmission of animal Influenza A viruses to humans. Further research conducted on the NP gene from various animal species and humans with respect to its host specificity could be useful for monitoring influenza A viruses.

None of the unique amino acids of NP lineages identified in this study is involved in RNA binding activities [10]. They are mainly correlated with host specificity of the viruses. Genetic analysis of the NP gene of the 12 swine influenza viruses has shown that the viruses display high nucleotide sequence identities similar to either Eurasian swine or classical swine viruses. Four potentially unique amino acids specific to Eurasian and classical swine lineages but not avian or human lineages have been identified at positions 350 (K/T), 371 (V/M), 444 (V/I), and 456 (L/V). In contrast, amino acids at positions 345 and 430 have been reported as amino acids unique to the classical swine lineage [23]. Two amino acids at positions 105 and 450 have been reported as amino acids specific for avian lineages [19]. However the research presented here has not established the amino acid at position 405 (405V) as highly correlated with the avian specific lineage as previously reported (Table 3) [3]. This study has also analyzed at least 5 amino acid positions (16, 283, 293, 372, and 422) unique to the human lineage indicating that 283P/283L are specific to human and avian lineages, respectively, as previously reported [24-26]. It has been known that the amino acid at position 16 is related to the N-terminal cleavage of the NP gene and correlated with the host specificity of the virus [27]. The amino acid motif of the NP gene of the human virus (ETD16G) is sensitive to host protease, while that of avian and swine viruses (ETG16G) is resistant [28,29]. Moreover, in this study, we were able to identify at least 5 amino acids of the NP gene (100, 217, 313, 316, and 425) unique to the pandemic H1N1 2009 viruses. Previous studies analyzed the NP gene of H1N1 2009 stored at the public database and the result showed that the amino acids V100 and V313 were highly conserved in the pandemic H1N1 2009 virus [30]. In addition, the tendency of a V to I mutation in NP100 has also been previously reported, similar to the finding in this study [26].

## Conclusion

In conclusion, our study provided the nucleotide sequences of the NP gene of 12 Thai swine influenza viruses of subtypes H1N1, H1N2 and H3N2. Phylogenetic and genetic analysis of the swine, avian and human influenza viruses confirmed the highly conserved nature of the NP gene within host-specific lineages. The NP gene of swine influenza viruses clustered with either Eurasian swine or classical swine viruses indicating the origins of the imported viruses. Unique amino acids specific to swine, avian and human influenza lineages were identified. This research highlights the significance of genetic variation of the NP gene from swine, avian and human influenza viruses in Thailand.

## Materials and methods

### Influenza A Virus from swine

The 12 swine influenza viruses in this study were isolated from swine raised in Thailand between 2005 and 2009. The viruses were obtained from swine farms in provinces of the central region (Saraburi, Ratchaburi and Nakhon Pathom) and eastern region (Chonburi and Chachoengsao) of Thailand. Virus isolation was performed as previously described [18]. The viruses were confirmed as influenza A virus by one-step realtime RT-PCR with primers and probe specific to the M gene. The viruses were then subtyped as H1N1 (n = 6), H1N2 (n = 1) and H3N2 (n = 5) by using primers specific to each subtype of swine influenza viruses (list of primers is available upon request). The viruses were propagated in Madin-Darby canine kidney (MDCK) cells in minimal essential medium (MEM) (Hyclone, USA) with 5% fetal calf serum (Hyclone) for 3 passages for further NP gene sequencing.

### Complete NP gene sequencing

Viral RNA was extracted from cell culture by using a QIAmp viral RNA mini kit (Qiagen, Hilden, Germany). cDNA synthesis of viral RNA and amplification of the NP gene by PCR were performed with specific primers with some modifications (Hoffman et al., 2001). In brief, cDNA synthesis was carried out by incubating the viral RNA with 0.5 ug of random primers at 70°C for 5 min and 4°C for 5 min. The mixture was added to 1× reaction buffer (Promega, Madison WI), 0.5 mM dNTPs, 2.5 mM MgCl2, 10 U of RNAsin Ribonuclease inhibitor and 1 U of ImProm-II Reverse Transcriptase and incubated at 25°C for 5 min, 42°C for 60 min and 70°C for 15 min. Amplification of the NP gene was carried out in 50 ul of PCR mixture by adding 4 ul of cDNA, 1× master mix (ReadyMix PCR master mix, Thermo Fisher Scientific, UK) and 0.5 umol of oligonucleotide primers specific to the NP gene. The amplification reaction included an initial denaturation step at 94°C for 3 min, followed by 40 cycles of denaturation at 94°C for 30 s, annealing at 55°C for 30 s and extension at 72°C for 30 s, and concluded by a final extension step at 72°C for 7 min. The PCR products were mixed with loading buffer (2% Orange G in 50% glycerol) and then separated by 1.5% agarose gel electrophoresis (FMC Bioproducts, Rockland, ME). PCR products of interest were purified by the QIAquick Gel Extraction Kit (Qiagen). DNA sequencing was carried out by dideoxynucleotide chain termination technique. Briefly, the sequencing reaction was performed using Big Dye Terminator V3.0 Cycle Sequencing Ready reaction (ABI, Foster city, CA) at a final volume of 20 ul containing 1× reaction dye terminator and 3.2 pmol of specific sequencing primers. The product of the sequencing reaction was analyzed in the ABI-Prism 310 Genetic Analyzer (Perkin Elmer, Norwalk, CT).

### Analysis of genetic variation of the NP gene of Swine influenza viruses

Nucleotide sequences were edited, validated and assembled by using Chromas version 1.45 (Technelysium Pty. Ltd., Australia), and SeqMan (DNASTAR, Madison, WI). The complete nucleotide sequences of the NP gene of influenza viruses from swine were submitted to the GenBank database with accession numbers shown in Table 1. Phylogenetic analyses were conducted in MEGA version 4 [31] using neighbor-joining method with Kimura 2-parameter. Bootstrap analysis was performed with 1000 replicates. The Bayesian tree was generated using the MrBayes V.3.1.2 [32] with 1 million generations using default heating parameters. The posterior probabilities were calculated to confirm tree topology. Genetic analyses for amino acid polymorphisms of the NP gene from viruses isolated from different host species were performed by amino acid alignments using the MegAlign program (DNASTAR). Additional NP nucleotide sequences from Thai seasonal H1N1 (n = 3), H3N2 (n = 8) and pandemic (H1N1) 2009 (n = 8) from humans as well as those from Thai HPAI (H5N1) from avian species (n = 24) and humans (n = 6) were included for phylogenetic and genetic analyses.

### Nucleotide substitution rates of the NP gene

Nucleotide substitution rates of the NP gene of swine, human and avian influenza A viruses recovered from 2003-2009 in Thailand were calculated using the computer program BEAST v1.4.7 applying the Bayesian Markov Chain Monte Carlo (BMCMC) [33]. Each nucleotide sequence was analyzed by codon-position-specific HKY+Γ substitution model as well as clock models (strict clock, uncorrelated relaxed clock and correlated relaxed clock). The BMCMC analysis was conducted with the parameters of at least 50 million states with 1000 sampling intervals and the 10% of each chain are 'burn-in' removed. The BMCMC analysis results were shown using Tracer V1.4.

## Competing interests

The authors declare that they have no competing interests.

## Authors' contributions

NT performed genome sequencing of the NP gene, phylogenetic analysis and drafted the manuscript. PK, RT SP and SD participated in virus isolation and drafting of the manuscript. DS conducted virus isolation. AT, YP and KS performed genetic and phylogenetic analyses. AA was responsible for experimental design, analyses and final approval of the manuscript. All authors read and approved the final manuscript.
